# The Depths from Skin to the Major Organs at Chest Acupoints of Pediatric Patients

**DOI:** 10.1155/2015/126028

**Published:** 2015-09-20

**Authors:** Yi-Chun Ma, Ching-Tien Peng, Yu-Chuen Huang, Hung-Yi Lin, Jaung-Geng Lin

**Affiliations:** ^1^Graduate Institute of Chinese Medicine, College of Chinese Medicine, China Medical University, Taichung 40402, Taiwan; ^2^Tai-An Hospital, Taichung 40402, Taiwan; ^3^Children's Hospital of China Medical University, Taichung 40402, Taiwan; ^4^School of Chinese Medicine, College of Chinese Medicine, China Medical University, Taichung 40402, Taiwan; ^5^Department of Medical Research, China Medical University Hospital, Taichung 40402, Taiwan

## Abstract

*Background*. Acupuncture is applied to treat numerous diseases in pediatric patients. Few reports have been published on the depth to which it is safe to insert needle acupoints in pediatric patients. We evaluated the depths to which acupuncture needles can be inserted safely in chest acupoints in pediatric patients and the variations in safe depth according to sex, age, body weight, and body mass index (BMI). *Methods*. We retrospectively studied computed tomography (CT) images of pediatric patients aged 4 to 18 years who had undergone chest CT at China Medical University Hospital from December 2004 to May 2013. The safe depth of chest acupoints was directly measured from the CT images. The relationships between the safe depth of these acupoints and sex, age, body weight, and BMI were analyzed. *Results*. The results demonstrated significant differences in depth among boys and girls at KI25 (kidney meridian), ST16 (stomach meridian), ST18, SP17 (spleen meridian), SP19, SP20, PC1 (pericardium meridian), LU2 (lung meridian), and GB22 (gallbladder meridian). Safe depth significantly differed among the age groups (*P* < 0.001), weight groups (*P* < 0.05), and BMI groups (*P* < 0.05). *Conclusion*. Physicians should focus on large variations in needle depth during acupuncture for achieving optimal therapeutic effect and preventing complications.

## 1. Introduction

Acupuncture has been used for thousands of years based on the theory of meridians and is crucial component of traditional Chinese medicine. Acupuncture has been applied to numerous diseases and has been found to be efficacious in treating chronic pain, postoperative nausea and vomiting, postoperative pain, chemotherapy-induced nausea, acute pain including dental pain, headache, hypertension, and chronic obstructive pulmonary disease (COPD), and seasonal allergic rhinitis [1, 2]. Acupuncture has also been applied to several conditions in children, such as postoperative nausea and vomiting [3], migraine and tension type headaches [4], chronic pain [5], allergic rhinitis [6, 7], asthma [8, 9], and nocturnal enuresis [10]. Thus, the safety of acupuncture is crucial.

Acupuncture may lead to severe complications, such as the transmission of diseases, needle fragments left in the body, nerve damage, pneumothorax, pneumoperitoneum, organ puncture, cardiac tamponade, and osteomyelitis [11]. Pneumothorax is the most frequently reported tissue or organ injury caused by acupuncture [12, 13]. Therefore, studying the needling depth and the distance from skin surface of chest acupoints to the major organs to decrease pneumothorax and cardiac tamponade is crucial.

Traditionally, the depth of needling at acupoints was measured by cun and has variations in ancient book, which has no difference between people of different body size. Owing to the fact that there is a lack of consistency in determining the depth of insertion, the depth of needle insertion was usually judged by clinical experience of acupuncture practitioners and the response from the patients. In children, it may be difficult to elicit a proper response to the needle insertion. Thus, it is critical to establish the data of acupoint depth for safe needling, specific for children.

A few surveys have been done by different measuring tools to investigate safe needle depth [14]. These studies have used autopsy, ultrasound, computed tomography (CT), or magnetic resonance imaging (MRI) as measurement methods and the results have varied among these detection methods. The inconsistent results might be caused by various measurement methods, ethnicity, age, sex, body size, medical conditions, and the definitions of safe needle depth. Depths measured according to images in living humans are greater than depths measured in cadavers, the tissues of which become dryer and smaller after freezing, anticorrosive positioning, and the dyeing process [14]. Hence, in this study, we used CT scan image, an appropriate measuring tool in surveying chest, to detect safe needle depth of chest acupoints in children.

Most previous studies have involved small sample sizes and adult groups. In pediatric groups, changes in muscle mass and fat volume and their distribution, particularly during the puberty stage, must be considered because of the influence on safe needle depth, which is unnecessary in adult groups. Scant studies on safe needle depth have been conducted in pediatrics. Only 2 previous studies, both conducted by Chen et al., have included pediatric groups; they measured the safe depth and therapeutic depth of abdomen acupoints according to CT scans [15, 16]. In our study, we included larger sample sizes and pediatric groups. We measured the safe depth of chest acupoints according to CT scans and evaluated the variations in safe depth according to sex, age, weight, and body mass index (BMI).

## 2. Methods

### 2.1. Study Population

All patients aged 4 to 18 years who had undergone a chest CT between December 2004 and May 2013 at China Medical University Hospital (CMUH) were selected. These patients had undergone CT scanning to evaluate acute chest condition, such as acute accidental injuries, pneumonia, pneumothorax, and cardiac diseases. Patients with chronic oncology diseases, chest-wall deformity, or chest-wall trauma were excluded because of the possible effect on the thickness of subcutaneous tissues or muscles of the chest-wall. Patients diagnosed with pneumothorax were measured on the contralateral side.

The age, sex, and body weight of each patient were obtained from chart records. BMI was measured according to weight (kg)/height^2^ (m^2^). Patients were divided into 5 groups according to age: 4 to 6 (48–83), 7 to 9 (84–119), 10 to 12 (120–155), 13 to 15 (156–191), and 16 to 18 (192–216) years (mo). Patients were also divided into 4 groups based on weight: below the third percentile, between the third and 85th percentiles, between the 85th and 97th percentiles, and at or above the 97th percentile, according to the new growth charts for Taiwanese children and adolescents [17]. Patients were divided into 4 groups based on BMI: underweight below the fifth percentile, healthy weight at or above the fifth percentile and below the 85th percentile, overweight at or above the 85th percentile, below the 95th percentile, and obesity at or above the 95th percentile, also according to the new growth charts [17, 18]. The data were analyzed anonymously. The Research Ethics Committee of CMUH approved this study.

### 2.2. Measurement of Safe Depth at Chest Acupoints

The acupoints were located according to a classical Chinese acupuncture technique, known as Tong Shen Cun (cun). The chest transverse Tong Shen Cun is one-eighth of the distance between the 2 nipples. The chest vertical Tong Shen Cun is 8 cuns of distance between CV22 to CV17. CV22 is located at the central point of 2 clavicles and the suprasternal notch. CV17 is located at the central point of the 2 nipples and on the sternum. The safe depths of acupoints in the conception vessel (CV) meridian (Renmai) were defined as the distance from the skin surface of the acupoint to the sternum, except CV22. The safe depth of CV22 was the distance from the skin to the trachea, and the safe depths of other acupoints over the chest were defined as the distance from the skin surface of acupoints to the pleura.

In total, 28 acupoints located at the conception vessel meridian (CV) (Renmai), kidney meridian (KI), stomach meridian (ST), spleen meridian (SP), gallbladder meridian (GB), lung meridian (LU), and pericardium meridian (PC) were measured. Patients diagnosed with pneumothorax were measured on the healthy side. From CV21 to CV16, the interval of each acupoint was 1.6 cuns. The kidney meridian, stomach meridian, and spleen meridian were 2, 4, and 6 cuns from the CV meridian. GB23 and GB22 were 7 and 8 cuns from CV17. LU2 was 6 cuns from CV21, and LU1 was the same distance from CV20. PC1 was 5 cuns from CV17.

The CT machines used at CMUH were the Light Speed16 CT Scanner (GE Healthcare, General Electric Company, USA), Optima Speed CT Scanner (GE Healthcare, General Electric Company, USA), and Optima CT660 (GE Healthcare, General Electric Company, USA). All chest CT images were captured in the transverse plane. The section thickness between each image was 5 mm. The safe depth was measured according to the CT image displayed on a Picture Archiving and Communication System (PACS) monitor (Realsync, Taiwan).

### 2.3. Statistical Analysis

The safe depths of acupoints among different groups of weight, age, and BMI were analyzed using one-way analysis of variance (ANOVA) or Brown-Forsythe test. Brown-Forsythe test was used when the variances were heterogeneous. Two-way ANOVA was then used to determine the effects considering the sex in the analysis of the other variables.

The generalized linear model was used to analyze whether sex, age, weight, and BMI are relevant factors in determining the safe depth of acupoints. Statistical analyses were performed using the SPSS software package, version 18.0 (SPSS Inc., Chicago, IL, USA) and *P* < 0.05 was considered significant.

## 3. Results

From December 2004 to May 2013, 319 patients (205 boys and 114 girls) ranging from 4 to 18 years of age were included in this study. All participants were Taiwanese. Among them, only 318 of those had weight data and 286 of those had height data to get BMI.  Table 1 provides the general characteristics of participants.

The mean and standard deviation of the safe depth for boys and girls for 28 chest acupoints are shown in Table 2. The shallowest depth was CV19 in boys and CV21 in girls. The greatest depth was LU2 in both sex groups. The deepest/shallowest ratio was 7 in boys and 5.7 in girls. Significant differences in depth among boys and girls were observed for KI25, ST16, ST18, SP17, SP19, SP20, PC1, LU2, and GB22. Among these acupoints, KI25, ST16, ST18, SP17, SP19, SP20, and PC1 in girls were deeper than those in boys. The LU2 and GB22 in boys were deeper than those in girls.

Safe depth significantly differed among age groups (*P* < 0.001) (Table 3). Age was a significant factor affecting safe depth. Safe depth significantly correlated with an increase in age only for KI23, ST17, LU2, SP17, and GB22. For another 22 chest acupoints, safe depth did not increase with age in the 10-to-12-year-old and older groups.

Safe depth significantly differed between weight groups (*P* < 0.05) (Table 4) and significantly increased with weight, except at the CV21 and CV22 acupoints. Safe depth in the above-97th-percentile group was 1.24 (CV22) to 2.11 (CV17) times deeper than the safe depth in the below-third-percentile group.

Safe depth also significantly differed between BMI groups (Table 5). An increase in the BMI was significantly correlated with the increase in safe depth, except CV21 and LU2. Safe depth in the obesity group was 1.23 (CV22) to 1.75 (CV22) times deeper than that in the underweight group.

We also applied generalized linear regression modeling to analyze whether age, sex, weight, and BMI are relevant factors in determining the safe depth of acupoints. The results revealed that sex and weight were the most crucial factors independent of age and BMI in determining the safe depth of chest acupoints (data not shown).

## 4. Discussion

This study used retrospective data and chest CT images from 2004 to 2013 in CMUH to estimate the variations in safe depth according to sex, age, weight, and BMI in children aged 4 to 18 years. We observed a significant difference in safe depth at KI25, ST16, ST18, SP17, SP19, SP20, PC1, LU2, and GB22 between both sexes, which significantly differed among the age groups, weight groups, and BMI groups.

In this study, the safe depth at KI25, ST16, ST18, SP17, SP19, SP20, and PC1 acupoints in girls was deeper than it was in boys. These acupoints were significantly deeper in girls around the breast. Thelarche in girls typically begins between the ages of 8 and 13 years, with an average onset age of 10.3 years [19]. Whereas the breast bud is one of the first signs of puberty, the estimated mean time for full breast development is 4.2 years [20]. Hence, breast development may cause a deeper safe depth of acupoints in girls. The pediatric sex difference in safe depth was noted around breast acupoints, which was not observable in abdominal acupoints [15]. This sex difference was also observed in adult chest acupoints [21, 22].

Safe depth significantly differed among age groups (*P* < 0.001). However, safe depth significantly correlated with an increase in age only in KI23, ST17, LU2, SP17, and GB22. At another 23 chest acupoints, safe depth did not increase with age in the 10-to-12-year-old and older groups. At each acupoint, depth increased significantly with age only before 12 years of age. During puberty, boys gain greater amounts of fat-free mass and skeletal mass, whereas girls acquire significantly more fat mass [23]. The mean age of puberty onset is approximately 10.5 years of age in girls and 11.5 years in boys. The normal range for pubertal onset is wide, which is between 8 and 12 years in girls and 9 and 14 years in boys. Pubertal timing is influenced by genetics, overall health, and environmental factors, such as social environment or endocrine disruptors. These factors may have contributed to the increase of fat or skeletal mass uncorrelated with age in the 10-to-12-year-old and older groups [24]. This may explain why safe depth increased significantly with age only before 12 years of age.

Safe depth significantly increased with weight, except at CV21 and CV22 acupoints. A thicker fat or muscle tissue layer in heavier children may explain this observation. This result is compatible with an adult study in which the safe depths in chest acupoints were related to body size [22]. At CV21 and CV22 acupoints, a thin layer of fat or muscle tissue was observable, which may be the cause of no differences being observed at these 2 points among the weight groups.

The BMI characterizes the relative proportion between the child's weight and height. The BMI is a valid predictor of adiposity and is therefore the optimal clinical standard for defining obesity in adults and in children older than 2 years of age [25]. Because BMI varies with race, age, sex, and pubertal stage, we used new growth charts for Taiwanese children and adolescents [17, 18]. In this study, safe depth significantly differed among BMI groups (Table 5). An increase in the BMI was significantly correlated with the increase in safe depth in most acupoints. A thicker fat tissue layer in obesity children can explain this result.

Generalized linear regression analysis revealed that sex and weight were relevant factors independent of age and BMI in determining the safe depth of chest acupoints. Previous studies had observed that BMI does not predict body fat very well in low to normal BMI children [26, 27]. Besides, large variation of the timing of puberty onset might cause complex conditions of fat tissue thickness or muscle size over the chest area. These could explain why BMI was less crucial than weight and sex in influencing safe depth over the chest.

This study is the first to investigate safe depth of chest acupoints in pediatric groups. We also studied factors such as sex, age, weight, and BMI that influence safe depth. However, our study has limitations, which include the retrospective design and the small sample size in the overweight group. In addition, our study participants were patients, not healthy children; however, we excluded patients with diseases that might affect the thickness of subcutaneous tissues or muscles of the chest-wall. Finally, because a single study may cause regional bias and all participants were Taiwanese children, international studies with a larger distribution should be conducted.

## 5. Conclusion

This study demonstrated that safe depths of chest acupoints around the breast were significantly deeper in girls. Safe depth increased significantly with age before 12 years of age. An increase in the weight and BMI was significantly correlated with the increase in safe depth. Thus, children with heavier weight would have deeper safe needle depth over chest acupoints. Physicians should focus on large variations of needle depth when performing acupuncture for achieving optimal therapeutic effects and for preventing complications. Future works about safe needle depth at acupoints over other body parts in children should be conducted and a formula considering weight or BMI index may be created for safe acupuncture.

## Figures and Tables

**Table 1:** General characteristics of subjects.

Characteristics	Subjects numbers
Gender	
Male	205
Female	114
Age (years)	
4–6	79
7–9	45
10–12	30
13–15	56
16–18	109
Weight	
<3rd percentile	32
3–85th percentile	215
85–97th percentile	42
≥97th percentile	29
BMI	
Underweight	52
Healthy weight	186
Overweight	18
Obesity	30

**Table 2:** Safe depth of 28 chest acupoints in different sex.

Acupoints	Boys	Girls	*P* value
	Mean ± S.D.	Mean ± S.D.	
CV22	22.39 ± 7.04	22.82 ± 7.20	0.61
CV21	5.86 ± 4.56	6.20 ± 4.70	0.54
CV20	6.14 ± 4.02	6.51 ± 4.12	0.44
CV19	5.73 ± 2.94	6.76 ± 4.22	0.11
CV18	6.34 ± 4.77	6.84 ± 4.08	0.35
CV17	6.75 ± 4.69	7.67 ± 5.02	0.10
CV16	9.48 ± 4.86	9.70 ± 5.53	0.72
KI27	27.50 ± 10.08	27.67 ± 9.56	0.88
KI26	17.15± 6.71	18.37 ± 8.20	0.15
KI25	15.66 ± 5.54	17.61 ± 7.28	0.008^*∗*^
KI24	14.75 ± 4.72	15.46±6.93	0.33
KI23	13.99 ± 4.78	14.23 ± 6.79	0.74
KI22	13.85 ± 5.05	14.08 ± 6.52	0.72
ST13	33.24 ± 13.83	34.57 ± 15.36	0.44
ST14	21.96 ± 8.27	22.72 ± 12.67	0.57
ST15	18.47 ± 7.57	20.50 ± 11.29	0.06
ST16	15.57 ± 6.21	17.75 ± 10.42	0.04^*∗*^
ST17	13.94 ± 5.27	14.95 ± 8.21	0.24
ST18	13.35 ± 4.82	14.96 ± 7.33	0.04^*∗*^
LU2	40.60 ± 17.10	35.07 ± 17.74	0.007^*∗*^
LU1	25.79 ± 10.16	26.40 ± 13.29	0.67
SP20	19.41 ± 7.28	22.29 ± 13.07	0.03^*∗*^
SP19	16.40 ± 6.00	19.31 ± 11.12	0.01^*∗*^
SP18	14.89 ± 5.36	15.34 ± 8.29	0.60
SP17	13.38 ± 3.96	14.99 ± 6.49	0.02^*∗*^
PC1	14.37 ± 4.61	16.54 ± 8.23	0.01^*∗*^
GB23	17.14 ± 5.95	16.38 ± 7.12	0.31
GB22	20.34 ± 8.15	17.46 ± 8.32	0.003^*∗*^

The unit of mean ± S.D. is mm.

^*∗*^Statistical significance.

**Table 3:** Safe depth of 28 chest acupoints in different age groups.

Points	4–6 y/o	7–9 y/o	10–12 y/o	13–15 y/o	16–18 y/o	*P* value^1^	*P* value^2^
	(*n* = 79)	(*n* = 45)	(*n* = 30)	(*n* = 56)	(*n* = 109)		
	Mean ± S.D.	Mean ± S.D.	Mean ± S.D.	Mean ± S.D.	Mean ± S.D.		
CV22	19.32 ± 4.84	21.28 ± 7.47	25.53 ± 8.80	24.04 ± 6.67	23.80 ± 7.16	<0.001^*∗*^	<0.001^*∗*^
CV21	3.84 ± 0.73	4.66 ± 1.36	7.71 ± 6.24	6.56 ± 6.37	7.30 ± 4.84	<0.001^*∗*^	<0.001^*∗*^
CV20	4.06 ± 2.03	5.97 ± 4.77	7.29 ± 4.59	6.50 ± 3.34	7.61 ± 4.38	<0.001^*∗*^	<0.001^*∗*^
CV19	4.40 ± 1.87	5.41 ± 1.79	6.61 ± 3.44	6.31 ± 2.75	7.36 ± 4.56	<0.001^*∗*^	<0.001^*∗*^
CV18	4.38 ± 1.34	5.67 ± 2.82	7.01 ± 3.18	6.57 ± 3.02	8.26 ± 6.47	<0.001^*∗*^	<0.001^*∗*^
CV17	5.25 ± 4.63	5.76 ± 2.89	8.16 ± 4.31	7.20 ± 4.02	8.59 ± 5.53	<0.001^*∗*^	<0.001^*∗*^
CV16	6.16 ± 2.69	8.10 ± 3.04	10.68 ± 4.85	10.00 ± 4.24	12.08 ± 6.00	<0.001^*∗*^	<0.001^*∗*^
KI27	19.23 ± 4.22	24.19 ± 6.34	32.03 ± 6.97	29.55 ± 7.23	32.74 ± 11.34	<0.001^*∗*^	<0.001^*∗*^
KI26	12.50 ± 3.74	16.34 ± 5.53	20.14 ± 6.92	18.07 ± 6.01	20.83 ± 8.39	<0.001^*∗*^	<0.001^*∗*^
KI25	12.04 ± 3.89	14.67 ± 4.16	18.51 ± 6.03	17.25 ± 5.82	19.14 ± 6.83	<0.001^*∗*^	<0.001^*∗*^
KI24	11.19 ± 2.98	12.77 ± 3.64	16.25 ± 5.93	16.10 ± 4.51	17.78 ± 6.29	<0.001^*∗*^	<0.001^*∗*^
KI23	10.82 ± 3.22	12.81 ± 3.71	14.50 ± 5.80	14.75 ± 4.83	16.50 ± 6.54	<0.001^*∗*^	<0.001^*∗*^
KI22	9.17 ± 2.32	12.10 ± 3.43	15.79 ± 4.70	15.24 ± 4.62	16.96 ± 6.17	<0.001^*∗*^	<0.001^*∗*^
ST13	22.10 ± 9.51	29.11 ± 10.75	40.46 ± 11.63	37.80 ± 10.44	40.08 ± 15.33	<0.001^*∗*^	<0.001^*∗*^
ST14	14.57 ± 4.45	19.01 ± 7.21	27.94 ± 11.44	24.11 ± 7.39	26.58 ± 11.04	<0.001^*∗*^	<0.001^*∗*^
ST15	12.54 ± 3.87	17.12 ± 4.85	23.50 ± 10.64	20.58 ± 7.41	22.98 ± 10.53	<0.001^*∗*^	<0.001^*∗*^
ST16	11.43 ± 5.34	14.83 ± 6.21	19.14 ± 9.78	17.90 ± 7.12	18.98 ± 8.53	<0.001^*∗*^	<0.001^*∗*^
ST17	10.79 ± 3.72	12.77 ± 4.45	15.18 ± 7.01	15.83 ± 6.47	16.46 ± 7.43	<0.001^*∗*^	<0.001^*∗*^
ST18	10.55 ± 3.66	12.29 ± 4.21	14.93 ± 5.12	14.82 ± 5.39	16.31 ± 6.87	<0.001^*∗*^	<0.001^*∗*^
LU2	26.07 ± 10.14	30.96 ± 14.09	41.36 ± 17.92	43.03 ± 14.33	47.86 ± 17.85	<0.001^*∗*^	<0.001^*∗*^
LU1	19.35 ± 4.65	23.31 ± 8.82	31.06 ± 12.67	26.52 ± 9.34	30.30 ± 13.56	<0.001^*∗*^	<0.001^*∗*^
SP20	13.54 ± 4.75	17.97 ± 6.92	26.46 ± 12.50	21.86 ± 7.49	24.08 ± 10.77	<0.001^*∗*^	<0.001^*∗*^
SP19	12.06 ± 4.75	15.67 ± 6.21	19.14 ± 9.78	17.90 ± 7.12	20.23 ± 9.25	<0.001^*∗*^	<0.001^*∗*^
SP18	9.56 ± 4.41	13.54 ± 5.10	17.51 ± 5.03	16.79 ± 5.42	18.09 ± 6.66	<0.001^*∗*^	<0.001^*∗*^
SP17	11.42 ± 2.60	12.40 ± 2.98	14.73 ± 3.65	14.93 ± 5.01	15.72 ± 6.42	<0.001^*∗*^	<0.001^*∗*^
PC1	10.84 ± 4.25	14.23 ± 4.37	17.76 ± 5.53	16.45 ± 5.89	17.26 ± 6.80	<0.001^*∗*^	<0.001^*∗*^
GB23	12.15 ± 4.24	14.80 ± 4.90	18.83 ± 5.48	18.43 ± 5.43	19.79 ± 6.73	<0.001^*∗*^	<0.001^*∗*^
GB22	13.48 ± 7.19	16.95 ± 7.10	20.40 ± 6.83	21.51 ± 7.45	23.08 ± 7.77	<0.001^*∗*^	<0.001^*∗*^

The unit of mean ± S.D. is mm.

^*∗*^Statistical significance.

^1^One-way ANOVA test or Brown-Forsythe test.

^2^Two-way ANOVA test, adjusted *P* value after controlling sex.

**Table 4:** Safe depth of 28 chest acupoints in different weight groups.

Points	<3rd	3rd–85th	85th–97th	≥97th	*P* value^1^	*P* value^2^
	(*n* = 32)	(*n* = 215)	(*n* = 42)	(*n* = 29)		
	Mean ± S.D.	Mean ± S.D.	Mean ± S.D.	Mean ± S.D.		
CV22	19.90 ± 4.73	22.06 ± 6.65	25.61 ± 7.49	24.82 ± 9.72	0.004^*∗*^	0.009^*∗*^
CV21	6.05 ± 6.26	5.42 ± 3.43	6.51 ± 3.44	9.32 ± 8.71	0.023^*∗*^	0.007^*∗*^
CV20	5.27 ± 3.47	5.68 ± 2.85	7.60 ± 5.33	9.85 ± 7.10	0.001^*∗*^	<0.001^*∗*^
CV19	5.26 ± 2.38	5.68 ± 2.55	6.44 ± 3.03	9.58 ± 7.30	0.001^*∗*^	<0.001^*∗*^
CV18	5.26 ± 2.26	6.14 ± 4.37	7.27 ± 3.92	9.64 ± 6.76	0.001^*∗*^	<0.001^*∗*^
CV17	5.53 ± 1.95	6.50 ± 3.86	8.10 ± 5.66	11.66 ± 8.39	<0.001^*∗*^	<0.001^*∗*^
CV16	7.60 ± 2.64	8.86 ± 4.02	11.13 ± 5.25	14.46 ± 9.31	<0.001^*∗*^	<0.001^*∗*^
KI27	24.26 ± 8.44	26.06 ± 7.65	32.02 ± 11.23	35.71 ± 16.43	<0.001^*∗*^	<0.001^*∗*^
KI26	15.38 ± 5.25	16.82 ± 6.44	19.86 ± 7.59	22.40 ± 11.39	0.001^*∗*^	<0.001^*∗*^
KI25	14.41 ± 3.42	15.87 ± 5.87	17.80 ± 6.65	20.09 ± 9.07	0.004^*∗*^	<0.001^*∗*^
KI24	13.40 ± 3.68	14.32 ± 4.90	17.09 ± 5.78	18.77 ± 9.09	0.001^*∗*^	<0.001^*∗*^
KI23	12.14 ± 4.10	13.48 ± 4.72	15.77 ± 5.47	18.18 ± 9.56	0.001^*∗*^	<0.001^*∗*^
KI22	12.06 ± 3.95	13.27 ± 4.90	15.56 ± 4.92	18.57 ± 9.36	<0.001^*∗*^	<0.001^*∗*^
ST13	30.03 ± 11.88	31.91 ± 12.10	36.90 ± 15.07	46.46 ± 22.73	<0.001^*∗*^	<0.001^*∗*^
ST14	18.53 ± 7.24	21.18 ± 8.96	25.46 ± 9.48	29.53 ± 15.82	<0.001^*∗*^	<0.001^*∗*^
ST15	14.66 ± 4.86	18.69 ± 8.43	21.36 ± 9.12	24.92 ± 13.61	0.001^*∗*^	<0.001^*∗*^
ST16	13.47 ± 5.26	15.67 ± 7.37	18.75 ± 8.53	21.10 ± 11.59	0.002^*∗*^	<0.001^*∗*^
ST17	12.09 ± 4.00	13.62 ± 5.38	15.51 ± 6.22	20.04 ± 11.70	<0.001^*∗*^	<0.001^*∗*^
ST18	12.32 ± 4.00	13.25 ± 4.87	15.15 ± 5.23	18.91 ± 10.94	0.001^*∗*^	<0.001^*∗*^
LU2	33.85 ± 15.00	36.76 ± 15.37	44.73 ± 18.54	48.32 ± 26.65	0.004^*∗*^	0.001^*∗*^
LU1	21.08 ± 6.39	24.65 ± 9.67	30.27 ± 11.52	35.40 ± 18.76	<0.001^*∗*^	<0.001^*∗*^
SP20	16.74 ± 5.89	19.60 ± 8.69	22.71 ± 10.46	27.40 ± 15.54	0.001^*∗*^	<0.001^*∗*^
SP19	14.81 ± 4.84	16.79 ± 7.73	18.99 ± 6.93	22.92 ± 13.58	0.003^*∗*^	<0.001^*∗*^
SP18	13.63 ± 4.74	14.08 ± 5.75	17.77 ± 5.40	19.77 ± 11.09	<0.001^*∗*^	<0.001^*∗*^
SP17	11.91 ± 2.24	13.43 ± 3.80	14.67 ± 3.60	19.05 ± 11.04	0.001^*∗*^	<0.001^*∗*^
PC1	14.00 ± 4.48	14.51 ± 5.50	16.15 ± 5.05	19.58 ± 11.10	0.006^*∗*^	<0.001^*∗*^
GB23	15.00 ± 4.08	15.90 ± 5.32	19.74 ± 6.51	21.71 ± 10.85	<0.001^*∗*^	<0.001^*∗*^
GB22	17.62 ± 6.86	18.23 ± 7.77	22.21 ± 7.62	24.63 ± 11.32	<0.001^*∗*^	<0.001^*∗*^

The unit of mean ± S.D. is mm.

^*∗*^Statistical significance.

^1^One-way ANOVA test or Brown-Forsythe test.

^2^Two-way ANOVA test, adjusted *P* value after controlling sex.

**Table 5:** Safe depth of 28 chest acupoints in different BMI groups.

Points	Underweight^a^	Healthy weight^b^	Overweight^c^	Obesity^d^	*P* value^1^	*P* value^2^
	(*n* = 52)	(*n* = 186)	(*n* = 18)	(*n* = 30)		
	Mean ± S.D.	Mean ± S.D.	Mean ± S.D.	Mean ± S.D.		
CV22	20.98 ± 7.51	21.89 ± 6.29	25.83 ± 7.26	25.88 ± 8.93	0.002^*∗*^	0.007^*∗*^
CV21	5.72 ± 4.97	5.53 ± 3.65	6.00 ± 2.86	8.93 ± 8.21	0.027^*∗*^	0.012^*∗*^
CV20	5.40 ± 3.07	5.65 ± 2.81	7.33 ± 3.91	9.46 ± 7.65	0.002^*∗*^	<0.001^*∗*^
CV19	5.34 ± 2.63	5.58 ± 2.36	6.39 ± 2.41	9.02 ± 6.81	0.001^*∗*^	<0.001^*∗*^
CV18	5.70 ± 2.32	6.11 ± 4.51	6.80 ± 4.06	9.52 ± 6.55	0.003^*∗*^	0.001^*∗*^
CV17	5.92 ± 2.69	6.46 ± 3.89	8.30 ± 6.06	10.24 ± 7.52	0.005^*∗*^	<0.001^*∗*^
CV16	8.09 ± 3.29	9.04 ± 3.99	9.32 ± 3.74	13.79 ± 8.70	<0.001^*∗*^	<0.001^*∗*^
KI27	25.48 ± 7.48	26.31 ± 7.48	31.33 ± 10.32	32.98 ± 15.00	0.007^*∗*^	0.002^*∗*^
KI26	16.59 ± 6.50	16.67 ± 5.86	20.07 ± 6.48	21.24 ± 11.00	0.017^*∗*^	0.003^*∗*^
KI25	15.57 ± 5.50	15.66 ± 5.51	17.83 ± 5.21	19.62 ± 9.35	0.022^*∗*^	0.007^*∗*^
KI24	13.79 ± 4.89	14.24 ± 4.65	15.87 ± 4.40	19.57 ± 8.90	<0.001^*∗*^	<0.001^*∗*^
KI23	12.88 ± 4.24	13.29 ± 4.72	14.24 ± 4.24	18.58 ± 9.03	<0.001^*∗*^	<0.001^*∗*^
KI22	12.96 ± 4.10	13.46 ± 4.74	14.45 ± 4.31	18.04 ± 9.57	0.004^*∗*^	<0.001^*∗*^
ST13	31.53 ± 11.14	32.41 ± 11.88	37.98 ± 13.91	39.61 ± 21.21	0.060	0.047^*∗*^
ST14	20.73 ± 8.47	21.10 ± 8.39	24.28 ± 8.32	28.03 ± 16.11	0.015^*∗*^	0.004^*∗*^
ST15	17.12 ± 7.33	18.32 ± 7.69	21.43 ± 7.25	24.12 ± 14.23	0.012^*∗*^	0.003^*∗*^
ST16	14.81 ± 7.10	15.36 ± 6.97	17.31 ± 5.68	21.52 ± 12.23	0.004^*∗*^	<0.001^*∗*^
ST17	12.69 ± 5.11	13.30 ± 5.27	14.84 ± 4.41	20.04 ± 11.31	<0.001^*∗*^	<0.001^*∗*^
ST18	13.01 ± 4.80	13.04 ± 4.76	14.25 ± 3.26	18.17 ± 10.06	0.002^*∗*^	<0.001^*∗*^
LU2	35.68 ± 13.72	36.56 ± 15.63	45.77 ± 19.37	45.00 ± 23.25	0.036^*∗*^	0.016^*∗*^
LU1	23.37 ± 8.77	24.53 ± 9.38	29.66 ± 9.15	33.19 ± 17.92	0.002^*∗*^	<0.001^*∗*^
SP20	18.30 ± 8.22	19.38 ± 8.36	21.41 ± 7.09	26.73 ± 15.71	0.005^*∗*^	0.001^*∗*^
SP19	15.96 ± 7.02	16.68 ± 7.31	17.65 ± 5.28	22.41 ± 13.00	0.011^*∗*^	0.003^*∗*^
SP18	13.62 ± 5.15	14.45 ± 5.38	15.86 ± 4.44	19.88 ± 11.66	0.003^*∗*^	<0.001^*∗*^
SP17	12.22 ± 2.98	13.55 ± 3.58	14.74 ± 3.86	17.54 ± 10.71	0.008^*∗*^	<0.001^*∗*^
PC1	14.22 ± 5.01	14.53 ± 5.33	14.71 ± 4.23	19.84 ± 10.88	0.004^*∗*^	<0.001^*∗*^
GB23	14.64 ± 4.44	16.37 ± 5.15	19.80 ± 6.06	21.46 ± 11.28	0.001^*∗*^	<0.001^*∗*^
GB22	16.94 ± 6.85	18.64 ± 7.24	22.26 ± 7.86	24.15 ± 11.59	0.002^*∗*^	<0.001^*∗*^

The unit of mean ± S.D. is mm.

^*∗*^Statistical significance.

^a^Underweight group: patients' BMI below the 5th percentile.

^b^Healthy weight group: patients' BMI at or above the 5th percentile and below the 85th percentile.

^c^Overweight group: patients' BMI at or above the 85th and below the 95th percentile.

^d^Obesity group: at or above the 95th percentile.

^1^One-way ANOVA test or Brown-Forsythe test.

^2^Two-way ANOVA test, adjusted *P* value after controlling sex.
